# Dataset on utilizing cropping system-based fertilization techniques to improve soil health and crop output while minimizing tillage

**DOI:** 10.1016/j.dib.2024.110385

**Published:** 2024-04-02

**Authors:** Md. Jahangir Alam, A.T.M. Anwarul Islam Mondol, Rabeka Sultana Smiriti, Mahammad Shariful Islam, Habib Mohammad Naser, Sanjida Akter, Zakaria Alam

**Affiliations:** a Soil Science Division, Bangladesh Agricultural Research Institute (BARI), Gazipur, Bangladesh; b Entomology Division, Bangladesh Rice Research Institute, Gazipur 1701, Bangladesh; c Tuber Crops Research Centre, BARI, Gazipur, Bangladesh

**Keywords:** Chemical fertilizer, Four crops cropping system, Rice equivalent yield, System productivity, Production efficiency Agricultural productivity, Soil nutrients

## Abstract

The dataset provided details on how tillage methods and nutrient management impacted the productivity of the four crops (mustard>mungbean>Transplanting (T.) aus >Transplanting (T.) aman) cropping system and the overall soil health. The specific tillage techniques examined were minimum tillage (MT), conventional tillage (CT), and deep tillage (DT). Regarding nutrient management, NM_1_ utilized 100 % soil test-based (STB) fertilization following fertilizer gradient generation (FRG); NM_2_ applied 125 % of STB after FRG-2018; NM_3_ consisted of 100 % STB (with 80 % from chemical fertilizers and 20 % from cow dung); and NM_4_ relied on native fertility without any fertilization. Over three consecutive seasonal years (2018–19, 2019–20, and 2020–21), twelve treatments were replicated three times following a factorial totally randomized design. The comparative analysis of crop yield, rice equivalent yield, system productivity and production efficiency indicated superior performance of MT over both CT and DT. Furthermore, in relation to agricultural productivity metrics, the application of the nutrition package NM_3_ demonstrated performance levels exceeding the average. The adoption of MT and the incorporation of the NM_3_ nutrition package led to notable advancements in organic matter, field capacity, microbial biomass nitrogen, microbial biomass carbon and soil nutrient levels (N, P, K, S, Zn, and B). Consequently, the synthesis of the NM_3_ with MT is posited as a strategic approach for soil enhancement and the augmentation of crop productivity.

Specification Table

**Table:** 

Subject	Agricultural and Biological Science
Specific subject area	Soil Science and crop science
Data format	Raw
Type of data	Tables
Data collection	The study obtained data under field conditions, utilizing a measuring scale and a weight machine to collect agronomic measurements. Additionally, information concerning soil physical, chemical, and biological aspects was acquired using laboratory equipment such as a laboratory oven, soil grinder, pressure plate apparatus, core sampler and core, hand penetrometer, soil hydrometer, pH meter, Kjeldahl machine, atomic absorption spectrophotometer, spectrophotometer, fume hood, and incubation chamber.
Data source location	The research site is situated in agro-ecological zone (AEZ) 28, located in the middle of the Madhupur tract. Positioned at an mean elevation of 8.4 meters above sea level, the experimental site is situated at approximately latitude 23° 59′ 14′′ N and longitude 90° 24′ 18” E.
Data accessibility	https://data.mendeley.com/datasets/rzhyyzx2z2/1
Related research article	Alam, M. J., Islam, M. S., Mondol, A. A. I., Naser, H. M., Salahin, N., Alam, M. K., ... & Alam, Z. (2024). Cropping system-based fertilizer strategies for crop productivity and soil health under minimum tillage in grey terrace soil. *Heliyon*.

## 1 Value of the Data

• The combination of minimum tillage with the 100 % soil test-based dose, incorporating 80 % chemical fertilizers and 20 % cow dung, results in improvements in the overall system productivity, production efficiency, crop yield, and soil health as evident in the dataset. When farmers implement this combined strategy, it has the capacity to promote sustainable and improved agricultural results. The collective influence of these practices has a positive impact on the agricultural system at the grassroots level.
• Researchers can capitalize on the chance to enhance field capacity, elevate levels of soil microbial biomass carbon, microbial biomass nitrogen, organic matter and enhance soil nutrient content through the utilization of this dataset. By preserving soil health, this integrated system becomes crucial in formulating highly intensive crop production plans and undertaking research on soil health.
• The dataset promotes the adoption of the integrated system as an approach that efficiently maximizes resources, demonstrating superior outcomes in terms of productivity and efficiency. This enables farmers to improve the efficient use of inputs such as fertilizers, tillage, and organic amendments, potentially resulting in more economically viable farming practices. Farmers can gather experience on crop accommodation throughout the year with the implementation of proper tillage.
• This dataset provides valuable insights for agricultural researchers, with a focus on enhancing crop yields and promoting sustainable farming practices. It elucidates the intricate connection between tillage methods, nutrient management, and crop productivity across various crops. The significant interactions observed among tillage, year, and nutrient management emphasize the necessity for tailored approaches to maximize agricultural output. The study underscores the effectiveness of integrating minimum tillage with specific nutrient management strategies to enhance crop performance. Furthermore, it investigates the impact of these practices on soil health indicators, which are fundamental for maintaining long-term soil fertility and productivity. Ultimately, this dataset can inform agricultural policies, refine farming methodologies, and facilitate the development of interventions to bolster food security and sustainability efforts.

## 2 Background

Meeting the food demand of an overgrowing population through excessive tillage and imprudent chemical fertilization under multiple cropping, especially puddling in rice fields, has degraded soil health, disrupted nutrient balance, and reduced crop productivity in the long run. For this perspective, it is time to overcome nutrient depletion of soil and conserve soil physical health due to the highly intensive cropping system**.** The dataset highlights the cropping system, productivity, and soil health issues utilizing one figure and three tables**.**

## 3 Data Description

**Fig. 1:**
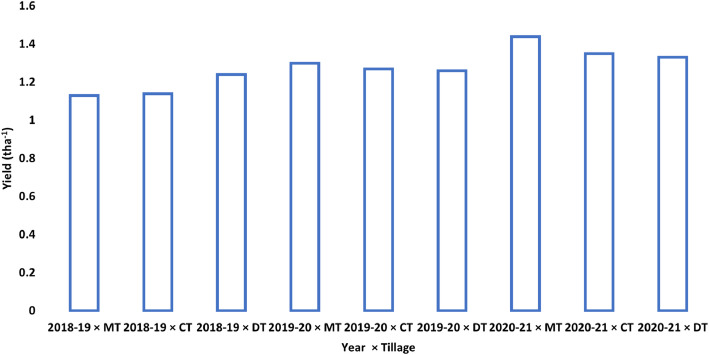
Interaction effect of year and tillage operation on average yield (t ha^−1^) of mustard. Where, MT=Minimum Tillage, CT=Conventional Tillage and DT=Deep Tillage.

There was no significant interaction between the year and tillage affecting the yield of Mungbean, T. aus, and T. aman. The combined effect of tillage and year was significant (*p* < 0.05) on the average yield of Mustard (Fig. 1). The MT demonstrated the highest mean yield (1.44 t ha^−1^), especially during 2020–21, trailed by CT (1.35 t ha^−1^), and DT (1.33 t ha^−1^).

The interaction between the year and nutrient management significantly (*p* < 0.05) influenced the yield of mustard, mungbean, and T. aus, with no such impact observed for T. aman (Table 1). The NM_3_ consistently exhibited the maximum average yield, particularly in 2020–21 (T. aus=3.96 t ha^−1^, T. aman=4.62 t ha^−1^, mungbean=1.40 t ha^−1^ and mustard=1.83 t ha^−1^), in comparison to other combinations.

**Table 1:** Interaction effect of year and nutrient management on average yield (t ha^−1^) of studied crops.

Year × Nutrient management	Mustard	Mungbean	T. aus	T. aman
2018-19 × NM_1_	1.29 e	0.88 e	3.17 d	3.75
2018-19 × NM_2_	1.33 de	0.92 de	3.46 bcd	3.76
2018-19 × NM_3_	1.48 bcd	1.00 cde	3.20 cd	4.22
2018-19 × NM_4_	0.59 f	0.68 f	2.65 e	2.78
2019-20 × NM_1_	1.46 cde	1.01 cd	3.32 cd	3.89
2019-20 × NM_2_	1.48 bcd	1.06 cd	3.64 abcd	4.00
2019-20 × NM_3_	1.66 ab	1.24 ab	3.65 abc	4.46
2019-20 × NM_4_	0.51 f	0.59 fg	2.56 e	2.59
2020-21 × NM_1_	1.60 bc	1.13 bc	3.47 bcd	4.07
2020-21 × NM_2_	1.61 bc	1.18 bc	3.82 ab	4.15
2020-21 × NM_3_	1.83 a	1.40 a	3.96 a	4.62
2020-21 × NM_4_	0.44 f	0.50 g	2.46 e	2.51
CV (%)	8.95	11.24	9.24	10.30
LS	***	***	**	NS

NM_1_ =100 % STB dose (chemical fertilizer), NM_2_ =125 % of STB dose (chemical fertilizer), NM_3_ =IPNS (80 % chemical fertilizer+ 20 % organic fertilizer), NM_4_ =Native fertility, ***=0.1 % level of significant, **=1 % level of significant, NS= Non significant, LS= Level of significant, CV= Co-efficient of variation, T. aus=Transplanting aus, T. aman=Transplanting aman

The interplay between tillage and nutrient packages demonstrated a considerable (*p* < 0.05) impact on all the examined crops (Table 2). In Mustard cultivation, the maximum mean yield was attained with MT when combined with NM_3_ (1.72 t ha^−1^), followed by DTNM_3_ (1.65 t ha^−1^) and CTNM_3_ (1.60 t ha^−1^). For mungbean, CT exhibited the highest average production when combined with NM_3_ (1.27 t ha^−1^), followed by DTNM_3_ (1.20 t ha^−1^) and MTNM_3_ (1.18 t ha^−1^). Regarding T. aus, the maximum mean yield was attained in DT (3.81 t ha^−1^) with NM_2_, followed by CTNM_2_, CTNM_3_, MTNM_3_, CTNM_1_, and MTNM_2_. In T. aman cultivation, CTNM_3_ resulted in the highest mean yield (4.66 t ha^−1^), followed by DTNM_3_ (4.46 t ha^−1^) and MTNM_3_ (4.18 t ha^−1^).

**Table 2:** Interaction effect of tillage and nutrient management on yield (t ha^−1^) of studied crops during 2018–19 to 2020–21.

Tillage operations × Nutrient management	Mustard	Mungbean	T. aus	T.aman
MT × NM_1_	1.48 bcd	1.07 bc	3.26 cd	3.85 b
MT × NM_2_	1.40 d	0.98 cd	3.36 abc	3.91 b
MT × NM_3_	1.72 a	1.18 ab	3.56 abc	4.18 ab
MT × NM_4_	0.56 e	0.56 c	2.82 de	2.95 c
CT × NM_1_	1.41 d	1.08 bc	3.43 abc	3.98 b
CT × NM_2_	1.54 abcd	1.04 bc	3.76 a	4.10 b
CT × NM_3_	1.60 abc	1.27 a	3.75 ab	4.66 a
CT × NM_4_	0.46 e	0.60 c	2.48 e	2.47 c
DT × NM_1_	1.46 cd	0.86 d	3.27 bcd	3.88 b
DT × NM_2_	1.48 bcd	1.13 abc	3.81 a	3.91 b
DT × NM_3_	1.65 ab	1.20 ab	3.50 abc	4.46 ab
DT × NM_4_	0.52 e	0.61 e	2.38 e	2.46 c
CV (%)	8.95	11.24	9.24	10.30
LS	*	***	**	*

MT=Minimum tillage, CT=Conventional tillage, DT= Deep tillage, NM_1_ =100% STB dose (chemical fertilizer), NM_2_ =125 % of STB dose (chemical fertilizer), NM_3_ =IPNS (80 % chemical fertilizer+ 20 % organic fertilizer), NM_4_ =Native fertility, ***=0.1 % level of significant, **=1 % level of significant, *=5 % level of significant LS= Level of significant, CV= Co-efficient of variation, T. aus=Transplanting aus, T. aman=Transplanting aman

Tillage and nutrient management exerted a significant influence on REY, SP, and PE, as indicated in Table 3, based on the crop yield in the third year. Regarding tillage, MT yielded significantly higher SP (9.93 t ha^−1^), REY (14.32 t ha^−1^) and PE (28.69 Kg ha^−1^ day^−1^) (*p* < 0.05). In terms of nutrient management, NM_3_ demonstrated superior performance, surpassing others with significant differences (SP=11.81 t ha^−1^, REY=17.46 t ha^−1^ and PE=34.14 Kg ha^−1^ day^−1^) (*p* < 0.05). The variables FC, BD, and PR did not exhibit significant differences with respect to tillage techniques but showed variations with nutrient management (Table 3). NM_3_ significantly produced the maximum FC (33.4 %), followed by BD (1.42 g cc^−1^) and PR (203 N cm^−2^) (*p* < 0.05). Tillage practices did not yield a significant impact on pH, OM, TN, TOC, K, MBC, P, S, Zn, and B (Table 3), except for MBN. The highest MBN value (19.1 µg g^−1^) was recorded during MT tillage practice, followed by CT and DT. Nutrient management had a consistent and significant impact on various soil parameters, with NM_3_ consistently demonstrating the highest values across pH, OM, TN, TOC, K, MBC, MBN, P, S, Zn, and B measuring at 6.13, 1.73 %, 0.091 %, 21.3 t ha^−1^, 0.17 meq 100 g^−1^, 288 µg g^−1^, 28.0 µg g^−1^, 21.2 µg g^−1^, 18.9 µg g^−1^, 1.69 µg g^−1^, and 0.28 µg g^−1^, respectively (*p* < 0.05).

**Table 3:** Mean value of REY, SP, PE, FC, BD, PR, soil pH, OM, TOC, MBC, TN, MBN, P, K, S, Zn and B after three years of tillage and nutritional management.

Tillage operations	REY (tha^−1^)	SP (tha^−1^)	PE (kg ha^−1^ day^−1^)	FC (%)	BD (g cc^−1^)	PR (N cm^−2^)	pH	OM	TN	TOC (t ha^−1^)	K (meq100 g^−1^)	MBC	MBN	P	S	Zn	B
											
								%				µg g^−1^					
MT	14.32 a	9.93 a	28.69 a	32.4	1.47	241	5.84	1.38	0.071	17.7	0.13	249	19.1 a	16.2	15.8	1.42	0.22
CT	13.99 ab	9.77 ab	28.25 ab	31.6	1.49	245	5.80	1.34	0.068	17.3	0.12	236	15.7 b	15.0	14.4	1.39	0.21
DT	13.46 b	9.37 b	27.07 b	31.5	1.46	234	5.81	1.32	0.062	16.8	0.12	227	15.0 b	14.8	14.2	1.38	0.20
CV (%)	5.06	5.25	5.25	3.12	1.43	9.37	3.75	7.48	15.27	7.06	11.8	10.9	15.7	10.3	14.7	14.4	11.4
LS	*	*	*	NS	NS	NS	NS	NS	NS	NS	NS	NS	**	NS	NS	NS	NS
Nutrient management																	
NM_1_	15.01 b	10.27 b	29.70 b	31.8 b	1.49 a	245 a	5.79 b	1.34 b	0.067 b	17.4 b	0.12 b	240 b	15.1 b	15.6 b	15.0 b	1.42 b	0.24 b
NM_2_	15.60 b	10.76 b	31.10 b	32.0 b	1.48 a	241 a	5.80 b	1.36 b	0.068 b	17.6 b	0.13 b	243 b	15.6 b	15.8 b	15.5 b	1.44 b	0.25 b
NM_3_	17.46 a	11.81 a	34.14 a	33.4 a	1.42 b	203 b	6.13a	1.73 a	0.091 a	21.3 a	0.17 a	288 a	28.0 a	21.2 a	18.9 a	1.69 a	0.28 a
NM_4_	7.63 c	5.90 c	17.09 c	30.2 c	1.51 a	270 a	5.52 c	0.96 c	0.041 c	12.7 c	0.07 c	179 c	7.8 c	8.7 c	9.7 c	1.03 c	0.08 c
CV (%)	5.06	5.25	5.25	3.12	1.43	9.38	3.75	7.48	15.27	7.06	11.8	10.9	15.71	10.3	14.7	14.3	11.4
LS	***	***	***	***	***	***	***	***	***	***	***	***	***	***	***	***	***

NM_1_ =100 % STB dose (chemical fertilizer), NM_2_ =125 % of STB dose (chemical fertilizer), NM_3_ =IPNS (80 % chemical fertilizer+ 20 % organic fertilizer), NM_4_ =Native fertility, MT=Minimum Tillage, CT=Conventional Tillage, DT=Deep Tillage, REY= Rice equivalent yield, SP= System productivity, PE= Production efficiency, FC= Field capacity, BD= Bulk density, PR= Penetration resistance, OM= Organic matter, TOC=Total organic carbon, MBC=Microbial biomass carbon, MBN=Microbial biomass nitrogen, TN=Total nitrogen, P= Phosphorus, K=Potassium, S=Sulphur, Zn=Zinc, B=Boron, *= 5 % level of significant, **=1 % level of significant, ***=0.1 % level of significant, NS= Non significant, LS= Level of significant, CV= Co-efficient of variation.

## 4 Experimental Design, Materials and Methods

### 4.1 Description and weather condition of the research location

Over the consecutive growing seasons spanning from 2018 to 2021, a study was conducted on the agricultural practices involving mustard (*Brassica campestris*), mungbean (*Vigna radiata*, T. aus (*Oryza sativa*, and T. aman (*Oryza sativa* in AEZ 28. This zone is located in the middle of Madhupur tract, with a mean elevation of 8.4 m above sea level. The experimental siteʼs coordinates are approximately 23° 59′ 14′′ N latitude and 90° 24′ 18” E longitude. The soil at the site, classified under the Chhiata Series, falls under the Aquepts suborder in the Inceptisols order of the USDA Soil Taxonomy. Specifically, it is categorized as Gleyic Alisols Cutanine and Gleyic Luvisols Cutanine within the Grey Terrace Soils Family [1,2]. The mean values of monthly temperature, precipitation, relative humidity and sunlight hours throughout a three-year cultivation period is outlined in Supplementary Fig. 1.

### 4.2 Experimental design and treatments

In this research, three different tillage methods were paired with four distinct nutrient management strategies. The tillage operations included deep tillage (DT) at a depth of 15–20 cm using two-wheel tractor firstly then used spade, conventional tillage (CT) at 8–10 cm depth using two-wheel tractors, and minimum tillage (MT) at 4–6 cm depth using two-wheel tractor operated seeder. Following the FRG [3] guidelines, the four nutrient management packages were as follows: NM_1_ = 100% STB (soil test-based) dose solely from chemical fertilizer, NM_2_ = 125% of STB dose solely from chemical fertilizer, NM_3_ = 100% STB dose (80% from chemical fertilizers and 20% from cow dung), and NM_4_ = Native fertility with no fertilization. A randomized complete block (RCB) design with a two-factor approach was employed to create twelve treatment combinations, and these combinations were distributed into three replications, resulting in a total of 36 plots. Each individual plot was measured to have dimensions of 5 m by 4 m. The process of sowing, transplanting, and engaging in intercultural operations for a four-crop cropping system was carried out in accordance with the guidelines outlined in the published article by Alam et al. [4].

### 4.3 Soil sampling, analysis and data collections

At the conclusion of the experiment, soil samples were collected from a depth of 0–15 cm, air-dried, and subsequently homogenized, pulverized, and sifted through a 2 mm sieve. These processed samples were then stored in plastic containers for future laboratory analysis. For example, the total N was quantified using a modified Kjeldahl method [5], while the available P was determined using a colorimetric approach [6]. The wet oxidation technique [7] was used to determine the organic matter content of the soil. The NH4OAC method [8] and the turbidimetric method [9] were used to calculate K and S, respectively. A pH meter with a glass electrode was used to measure pH [10]. Micronutrients (Zn and B) were investigated using an atomic absorption spectrophotometer. For estimating the bulk density, a core sampler was used [11]. The soil penetrating resistance is the force needed to push a metal rod with a specific tip through soil was measured using the hand penetrometer Eijkelkamp (Netherlands). The hand penetrometer was used in the experimental plot through pushing the the metal rod with 1 number cone (base area of cone was 1 cm^2^) then took the manometer reading. Then soil penetration resistance was calculated by dividing the manometer reading with base area of 1 number cone. Microbial biomass C was assessed using the chloroform fumigation-incubation method [12]. In this process, 40 g of soil, maintained at 55% of its water-holding capacity, were placed in 50 ml glass beakers. After a day of fumigation, the soil was extracted and subsequently incubated with 10 ml of 1 N potassium hydroxide (KOH) for ten days at a temperature of 25 °C. The production of carbon dioxide was determined by titrating KOH with 1 N hydrochloric acid (HCl). [13]. The calculation for soil microbial biomass carbon involved multiplying the milligrams of CO2-C generated per kilogram of fumigated soil by an efficiency factor of 0.41 [14]. Microbial biomass nitrogen was determined following the procedure outlined by Brookes et al. [15]. The total organic carbon (TOC) and organic carbon (OC) were calculated using the procedure described by Alam et al. [4]. Upon reaching harvesting maturity, the harvesting process was carried out using the method outlined by Alam et al. [16,17] to convert the crop yield into per hectare of land. The system productivity (SP), rice equivalent yield (REY) and production efficiency (PE) were computed according to the procedure of Alam et al. [4]. The calculations for REY, SP, and PE were derived from the crop production statistics of the third year.

Before commencing the experiment, nine soil samples were gathered from the field representing the entire experimental plot. These samples were combined to create a composite sample, which was then divided into three portions for analysis. The results for the physical and chemical properties of these soil samples were averaged (Supplementary Table S1). Similarly, decomposed cow dung was dried and divided into three samples for nutrient analysis. The values obtained from these three samples of decomposed cow dung are presented as range (Supplementary Table S2).

### 4.4 Statistical analysis

A three-year combined analysis and separate annual analyses of variance (ANOVA) were conducted using the open-source R software [18]. Mean values of treatment combinations were compared utilizing Tukey's Honestly Significant Difference (HSD) test at a significance level of *p* < 0.05.

## Limitations

The dataset exclusively encompasses data pertaining to individual crop yield, cropping system productivity, and soil parameters; however, it does not include physiological data or economic analyses.

## Ethics Statement

All authors have read and follow the ethical requirements for publication in Data in Brief and our work meets these requirements. Our work does not involve studies with animals and humans.

## CRediT authorship contribution statement

**Md. Jahangir Alam:** Writing – review & editing, Writing – original draft, Validation, Supervision, Project administration, Methodology, Investigation, Conceptualization. **A.T.M. Anwarul Islam Mondol:** Supervision, Investigation. **Rabeka Sultana Smiriti:** Formal analysis. **Mahammad Shariful Islam:** Supervision, Investigation. **Habib Mohammad Naser:** Supervision, Investigation. **Sanjida Akter:** Formal analysis. **Zakaria Alam:** Writing – review & editing, Visualization, Formal analysis, Data curation.

## Data Availability

Dataset on utilizing cropping system-based fertilization techniques to improve soil health and crop output while minimizing tillage (Original data) (Mendeley Data).

## Appendix Supplementary materials

Image, application 1

## References

[1] Huq S.M.I., Shoaib J.U.Md. (2013).

[2] WRB I.-W. (2006). World reference base for soil resources. World Soil Resour. Rep..

[3] (2018).

[4] M.J. Alam, M.S. Islam, A.A.I. Mondol, H.M. Naser, N. Salahin, M.K. Alam, M.M. Islam, S. Akter, Z. Alam, Cropping system-based fertilizer strategies for crop productivity and soil health under minimum tillage in grey terrace soil, Heliyon (2024). https://www.cell.com/heliyon/pdf/S2405-8440(24)00137-3.pdf (Accessed 6 February 2024). 10.1016/j.heliyon.2024.e24106 PMC10806292 38268576

[5] Page A.L., Miller R.H., Kenney D.R. (1989).

[6] Murphy J., Riley J.P. (1962). A modified single solution method for the determination of phosphate in natural waters. Anal. Chim. Acta.

[7] M.L. Jackson, Soil chemical analysis, Pentice hall of India Pvt, Ltd New Delhi India 498 (1973) 151–154.

[8] Hanlon E.A., Johnson G.V., Bray/Kurtz Mehlich (1984). AB/D and ammonium acetate extractions of P, K and MG in four oklahoma soils. Commun. Soil Sci. Plant Anal..

[9] Sperber I. (1948). A direct turbidimetric method for determining ethereal sulfates in urine. J. Biol. Chem..

[10] M.L. Jackson, Soil Chemical Analysis Prentice Hall, Inc Englewood Cliffs NJ 498 (1958) 183–204.

[11] Karim Z., Rahman S.M., Ali M.I., Karim A. (1988).

[12] Jenkinson D.S., Powlson D.S. (1976). The effects of biocidal treatments on metabolism in soil—V: a method for measuring soil biomass. Soil Biol. Biochem..

[13] Anderson J.P.E., Page A.L., Page A.L. (2015). Soil Respiration.

[14] Voroney R.P., Paul E.A. (1984). Determination of kC and kNin situ for calibration of the chloroform fumigation-incubation method. Soil Biol. Biochem..

[15] Brookes P.C., Landman A., Pruden G., Jenkinson D.S. (1985). Chloroform fumigation and the release of soil nitrogen: a rapid direct extraction method to measure microbial biomass nitrogen in soil. Soil Biol. Biochem..

[16] Z. Alam, S. Akter, M.A.H. Khan, M.S. Alam, S. Sultana, S. Akhter, M.M. Rahman, M.M. Islam, Yield performance and trait correlation of BARI released sweet potato varieties studied under several districts of Bangladesh, Heliyon 9 (2023). https://www.cell.com/heliyon/pdf/S2405-8440(23)05411-7.pdf (Accessed 24 October 2023). 10.1016/j.heliyon.2023.e18203 PMC10372318 37519685

[17] Alam Z., Akter S., Khan M.A.H., Amin M.N., Karim M.R., Rahman M.H.S., Rashid M.H., Rahman M.M., Mokarroma N., Sabuz A.A., Alam M.J., Roy T.K., Rahaman E.H.M.S., Ali M.A., Chanda D., Sarker U. (2024). Multivariate analysis of yield and quality traits in sweet potato genotypes (Ipomoea batatas L.). Sci. Hortic..

[18] R studio, R: The R Project for Statistical Computing, (2020). https://www.r-project.org/(Accessed 2 November 2023).

