# Supplementary material for: Dataset on utilizing cropping system-based fertilization techniques to improve soil health and crop output while minimizing tillage
Source: Data Brief. 2024 Apr 2;54:110385. doi: 10.1016/j.dib.2024.110385 (PMC11016950; doi:10.1016/j.dib.2024.110385)
Supplement: Supplementary file 1 [file mmc1.docx]

Supplementary Table S1. Physico-chemical properties of initial soil during 2018

| Physical properties | | | | | | | | |
| --- | --- | --- | --- | --- | --- | --- | --- | --- |
| Soil depth (cm) | Particle size (%) | | | Textural class | | | | |
| 0-15 | Sand | Clay | Silt | Clay loam | | | | |
|  | 42.28 | 28.44 | 29.28 |  |  |  |  |  |
|  | Moisture content (%) | | | | Bulk density  (g cm^-1^) | Penetration resistance  (N cm^-2^) | | |
|  | 0.3 bar (FC) | 1.0 bar | 2.0 bar | 3.0 bar |  |  |  |  |
|  | 30.20 | 24.89 | 21.34 | 19.12 | 1.51 | 267 | | |
| Chemical properties | | | | | | | | |
| Soil depth (cm) | pH | OM | Total N | P  (µg g^-1^) | K  (meq 100 g^-1^) | S | Zn | B |
|  |  | % | |  |  | µg g^-1^ | | |
| 0-15 | 5.54 | 1.14 | 0.058 | 12.3 | 0.12 | 11.9 | 1.19 | 0.15 |
| Critical level | - | - | - | 7.0 | 0.12 | 10 | 0.60 | 0.20 |
| Interpretation | Acidic | Low | Very Low | Low | Low | Low | Low | Low |

Supplementary Table S2. Nutrient content of decomposed cowdung

| **Item** | **OM** | **N** | **P** | **K** | **S** | **Zn** | **B** |
| --- | --- | --- | --- | --- | --- | --- | --- |
|  | **%** | | | | | | |
| Cowdung | 9.1-10.5 | 0.817.9-8.3 | 0.79-0.95 | 0.51-0.55 | 0.3-0.4 | 0.13-0.17 | 0.010.012 |


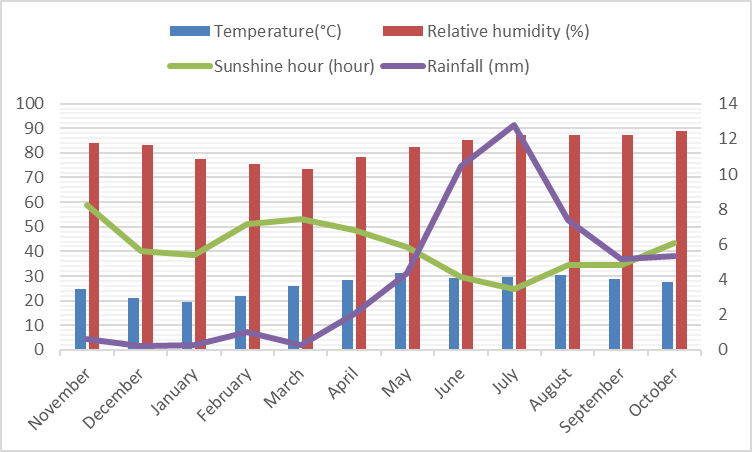


Supplementary Figure 1. Monthly average temperature, relative humidity, sunshine hour and rainfall in experimental area at crop growing period (2018-2021).
